# The Diabetic Hand as a Diagnostic Blind Spot: A Case of Severe Pseudohyperglycemia Masking Critical Hypoglycemia

**DOI:** 10.7759/cureus.104040

**Published:** 2026-02-21

**Authors:** King Hei Stanley Lam, Yonghyun Yoon, Daniel Chiung-Jui Su, Teinny Suryadi, Ngai Ho Yin Allen, Anwar Suhaimi, K. Dean Reeves

**Affiliations:** 1 Faculty of Medicine, The Chinese University of Hong Kong, New Territories, HKG; 2 Faculty of Medicine, The University of Hong Kong, Hong Kong, HKG; 3 The Board of Clinical Research, The Hong Kong Institute of Musculoskeletal Medicine, Kowloon, HKG; 4 Orthopedic Surgery, Hallym University Kangnam Sacred Heart Hospital, Seoul, KOR; 5 Orthopaedics, Incheon Terminal Orthopedic Surgery Clinic, Incheon, KOR; 6 Physical Medicine and Rehabilitation, Chi Mei Medical Center, Tainan, TWN; 7 Physical Medicine and Rehabilitation, Synergy Clinic, Jakarta, IDN; 8 Physical Medicine and Rehabilitation, Hermina Podomoro Hospital, Jakarta, IDN; 9 Medical Education, Hong Kong Institute of Musculoskeletal Medicine, Kowloon, HKG; 10 Rehabilitation Medicine, University Malaya Medical Centre, Kuala Lumpur, MYS; 11 Rehabilitation Medicine, University Malaya, Kuala Lumpur, MYS; 12 Rehabilitation Medicine, Private Practice, Kansas City, USA

**Keywords:** blood glucose monitoring, capillary blood glucose, carpal tunnel syndrome, diabetic hand, diagnostic errors, hand osteoarthritis, hospital survey on patient safety culture (hsopsc), patient’s safety, point-of-care testing, pseudohyperglycemia

## Abstract

Carpal tunnel syndrome (CTS) and hand osteoarthritis (OA) are prevalent, painful comorbidities in diabetes mellitus. While their symptomatic burden is recognized, their potential to create critical diagnostic blind spots by compromising point-of-care glucose monitoring is an underappreciated patient safety hazard. These conditions can compromise the local capillary bed through nerve compression, joint inflammation, and microvascular dysfunction, thereby invalidating the core physiological assumption of point-of-care glucose monitoring: that a fingertip sample accurately reflects systemic glucose levels.

An 80-year-old woman with type 2 diabetes presented for knee surgery. A routine postoperative capillary blood glucose (CBG) check yielded a value of 23 mmol/L, triggering a protocol for insulin administration. As the patient was asymptomatic, clinical suspicion led to a venous blood glucose test, which revealed hypoglycemia at 2.5 mmol/L. A systematic, multi-step verification was performed: the device was recalibrated, a repeat CBG from a different site on the same hand was obtained, and a second glucometer of a different brand was used to test both the ipsilateral and contralateral hands. All point-of-care readings remained persistently elevated (23-24 mmol/L), confirming a reproducible physiological error. A subsequent physical examination identified previously undiagnosed bilateral CTS and severe hand OA, pathologies constituting a "diabetic hand" with compromised peripheral perfusion.

This near-miss event presents a critical paradox: severe pseudohyperglycemia in a setting where classic physiology predicts the opposite artifact. The finding persisted despite the exclusion of common technical errors, underscoring a novel and dangerous failure mode associated with the compromised diabetic hand. The case demonstrates that such hand syndromes can catastrophically invalidate point-of-care glucose monitoring. It argues for the routine assessment of hand pathology in diabetic patients and warrants immediate venous verification of any clinically discordant point-of-care result to prevent catastrophic error.

## Introduction

The concept of the "diabetic hand" encompasses a spectrum of musculoskeletal and neuropathic disorders, serving as a local manifestation of systemic disease [1, 2]. This notion is supported by evidence linking specific hand abnormalities, such as limited joint mobility, to the presence of other microvascular complications in diabetes [3, 4]. Among the most prevalent and painful components of this syndrome are carpal tunnel syndrome (CTS) and hand osteoarthritis (OA). CTS is a well-documented comorbidity in diabetes, with a pathophysiology intrinsically linked to underlying neuropathy and microvascular dysfunction [5, 6]. Similarly, hand OA frequently coexists with type 2 diabetes, sharing inflammatory and metabolic pathways [7-9].

Under normal physiological conditions, capillary blood glucose (CBG) closely approximates arterial glucose levels because capillaries are highly permeable to glucose, allowing rapid equilibration between blood and interstitial fluid [10]. This equilibrium forms the basis for point-of-care testing: a fingertip capillary sample is assumed to reflect systemic glucose concentrations accurately. However, when peripheral perfusion is compromised, as in shock, severe peripheral vascular disease, or Raynaud's phenomenon, sluggish capillary flow allows prolonged tissue contact with blood, resulting in increased glucose extraction by metabolically active tissues. This produces artifactually low capillary readings relative to arterial or venous levels, a well-documented phenomenon termed pseudohypoglycemia [11, 12]. Thus, in any hypoperfused extremity, the expected direction of error is toward falsely low glucose measurements.

While the symptomatic management of CTS and hand OA is a recognized focus of pain medicine [5, 6], their potential to create critical diagnostic blind spots by compromising routine metabolic monitoring is less appreciated. CBG testing is a cornerstone of diabetes management, yet its accuracy is predicated on obtaining a reliable sample from a well-perfused capillary bed [10]. Compromised tissue perfusion represents a significant and often overlooked source of pre-analytical error in point-of-care testing [10]. The complex local pathophysiology of the advanced diabetic hand, characterized by nerve compression, joint inflammation, microvascular dysfunction, and an altered interstitial environment, may perturb the relationship between capillary and systemic glucose in ways that are not currently understood.

This case report presents a critical near-miss event that illustrates an unexpected variation from this physiological rule. We describe a patient with previously undiagnosed bilateral CTS and severe hand OA in whom a grossly elevated CBG reading (23 mmol/L) was discordant with an asymptomatic clinical state and subsequently found to represent undetected hypoglycemia (venous glucose 2.5 mmol/L) [13]. This observation contradicts the established expectation that hypoperfused extremities produce pseudohypoglycemia [11, 12]. The purpose of this report is to document this unexpected discrepancy, describe the systematic investigation undertaken, and generate hypotheses for future study. Current standards for inpatient diabetes management emphasize the importance of accurate glucose monitoring and appropriate insulin administration protocols [14, 15].

## Case presentation

An 80-year-old woman with a longstanding history of type 2 diabetes was admitted for elective knee arthroscopy to manage a symptomatic effusion secondary to severe tibiofemoral OA (Kellgren-Lawrence grade 3-4) and calcium pyrophosphate crystal deposition disease (pseudogout). Her diabetes was managed with oral agents (sitagliptin and metformin), supplemented with a sliding-scale insulin regimen for anticipated postoperative glycemic fluctuations.

The patient's most recent HbA1c, measured one month prior to admission, was 6.7% (49 mmol/mol). Admission hematocrit was 38%, within the normal range. Renal function was normal (creatinine 0.8 mg/dL (70.7 μmol/L), eGFR >60 mL/min/1.73 m²). The patient had received no dextrose-containing intravenous fluids in the preceding 12 hours. Her last insulin dose was six units of regular insulin administered subcutaneously approximately 14 hours prior to the event, as part of a "sliding scale" regimen that had not been triggered earlier due to stable glucose readings. The patient's last oral intake was a standard hospital diabetic meal five hours prior to the CBG check. She had been NPO (nil per os) for procedures earlier in the day but had resumed oral intake by evening.

On the third postoperative night, approximately five hours after her evening meal, a routine CBG check from a fingertip on her right hand returned a value of 23 mmol/L, a reading indicative of severe hyperglycemia. This triggered the standard nursing protocol to prepare a corrective insulin dose, which was held pending review. However, the attending physician was alerted to a profound clinical dissonance: the patient was asleep, entirely asymptomatic, and displayed no signs of hyperglycemic crisis such as polyuria, polydipsia, or dehydration. Given this incongruity and the patient's age, a population at heightened risk for hypoglycemia complications, an urgent laboratory venous blood glucose (VBG) test was ordered prior to any intervention, with results available within 15 minutes.

The VBG result revealed the true metabolic state: a plasma glucose level of 2.5 mmol/L, representing critical, life-threatening hypoglycemia. To investigate this discrepancy, an immediate point-of-care verification protocol was initiated. First, the original glucometer was recalibrated. A repeat CBG from a different fingertip on the same (right) hand was performed, yielding an unchanged result of 23 mmol/L. Second, a glucometer of a different brand was used to test the same puncture site, confirming an identical reading. Finally, to exclude a unilateral artifact, both glucometers were used to test a single puncture site on a fingertip of the contralateral left hand, with both devices returning a similarly elevated reading of 24 mmol/L. This systematic, multistep verification confirmed a persistent and reproducible physiological error, independent of the specific device or hand tested. A complete chronological record of all glucose measurements is provided in Table 1.

**Table 1:** Sequential Capillary, Venous, and Continuous Glucose Monitoring Measurements Glucometers A and B are different-brand point-of-care devices, both calibrated prior to use, ruling out device malfunction. Freestyle Libre (Abbott), placed on the upper arm, measures interstitial glucose at a site remote from the affected fingertips. Venous samples analyzed by the hospital laboratory serve as the gold standard reference. The initial measurement at Day 3, 24:00 (midnight) was obtained five hours postprandial, with all verification measurements completed within 25 minutes (24:05-24:25). Day 4-5 measurements were obtained during the 48-hour stabilization period, with follow-up one month post-HD. The "Difference from VBG" column quantifies overestimation compared to venous glucose. Values ≤ +0.6 mmol/L (arm and Libre) indicate clinically acceptable accuracy; values > +4.0 mmol/L (fingerstick) represent clinically significant pseudohyperglycemia that could lead to inappropriate insulin administration. Key observations: (1) Site specificity, arm readings on Day 4 were within +0.2-0.3 mmol/L of venous glucose while simultaneous fingerstick readings showed +8.4-8.7 mmol/L overestimation, confirming artifact localization to the fingertips; (2) CGM correlation, Libre readings were consistently within +0.4-0.6 mmol/L of venous glucose; (3) Initial discrepancy, fingerstick on Day 3 overestimated venous glucose by +20.5-21.5 mmol/L; (4) Progressive improvement, discrepancy decreased to +4.7 mmol/L by Day 5; (5) Post-HD effect, discrepancy improved to +1.5 mmol/L after nerve decompression, suggesting a contributing role for median nerve compression. The persistent, reproducible artifact across two devices and both hands confirms a patient-specific physiological phenomenon localized to the fingertips, with partial reversibility following nerve decompression providing preliminary evidence supporting a causal role. HD, ultrasound-guided hydrodissection of the median nerve; VBG, venous blood glucose.

Time	Sampling Site	Device	Glucose Reading (mmol/L)	Venous Glucose (mmol/L) at the Same Time	Difference from VBG
Day 3, 24:00	Right hand, fingertip 1	Glucometer A (original)	23.0	2.5	+20.5
Day 3, 24:05	Venous sample	Laboratory	—	2.5	—
Day 3, 24:20	Right hand, fingertip 2	Glucometer A (recalibrated)	23.0	2.5	+20.5
Day 3, 24:22	Right hand, same fingertip	Glucometer B (different brand)	23.0	2.5	+20.5
Day 3, 24:24	Left hand, fingertip	Glucometer A	24.0	2.5	+21.5
Day 3, 24:25	Left hand, same fingertip	Glucometer B	24.0	2.5	+21.5
Day 4, 08:00	Right hand, fingertip	Glucometer A	15.2	6.8	+8.4
Day 4, 08:00	Left hand, fingertip	Glucometer A	15.5	6.8	+8.7
Day 4, 08:00	Right proximal arm	Glucometer A	7.1	6.8	+0.3
Day 4, 08:00	Right medial arm	Glucometer A	7.0	6.8	+0.2
Day 4, 08:00	Upper arm	Freestyle libre	7.3	6.8	+0.5
Day 4, 14:00	Right hand, fingertip	Glucometer A	16.8	10.8	+6.0
Day 4, 14:00	Upper arm	Freestyle libre	11.4	10.8	+0.6
Day 4, 20:00	Right hand, fingertip	Glucometer A	15.5	9.8	+5.7
Day 4, 20:00	Upper arm	Freestyle libre	10.2	9.8	+0.4
Day 5, 02:00	Right hand, fingertip	Glucometer A	16.5	4.2	+12.3
Day 5, 02:00	Upper arm	Freestyle libre	4.6	4.2	+0.4
Day 5, 08:00	Right hand, fingertip	Glucometer A	9.8	5.1	+4.7
Day 5, 08:00	Upper arm	Freestyle libre	5.6	5.1	+0.5
1 month post-HD	Right hand, fingertip	Glucometer A	7.2	5.7	+1.5
1 month post-HD	Upper arm	Freestyle libre	6.1	5.7	+0.4

This critical discrepancy, which averted a potentially fatal iatrogenic insulin administration, prompted an immediate and focused physical examination of both upper extremities. Upon specific questioning prompted by this event, the patient reported a chronic, poorly characterized history of nocturnal hand numbness and tingling that she had not previously mentioned. The examination revealed previously undocumented pathology consistent with advanced "diabetic hand" syndrome and OA of the hands.

Neurological findings

Bilateral positive Tinel's sign (tingling upon percussion over the median nerve at the wrist) and Phalen's sign (symptoms reproduced with sustained wrist flexion). Thenar muscle atrophy was present bilaterally, more pronounced on the left.

Musculoskeletal findings

Advanced OA in multiple finger joints, characterized by palpable Heberden's and Bouchard's nodes, reduced range of motion, and bony enlargement. The patient's hand pathology was quantified where possible. Retrospective review of prior hand radiographs (reports only from the electronic records system of the government hospital), which the patient disclosed she had from follow-up at a government hospital orthopedic department, confirmed right-hand OA Kellgren-Lawrence grade 4 and left-hand grade 3. Ultrasound examination performed in the outpatient clinic following discharge revealed marked median nerve enlargement at the wrist (cross-sectional area 22 mm² on the right and 28 mm² on the left; normal <10 mm²). No nerve conduction studies were performed during the admission. Notable dermatological changes included skin that was notably thickened and stiff, with palpable subcutaneous fibrosis and reduced palmar extension.

Vascular inspection

**Figure 1:**
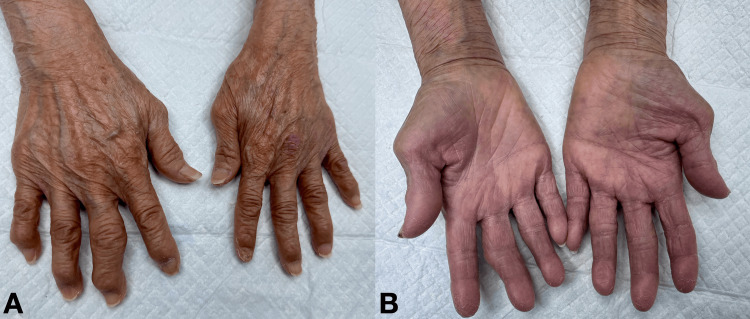
Clinical Photographs Demonstrating Findings of Advanced Osteoarthritic and Diabetic Hand (A) Dorsal view of the patient's hands and wrists demonstrating characteristic deformities of advanced osteoarthritis, including prominent Heberden's nodes (distal interphalangeal joints) and Bouchard's nodes (proximal interphalangeal joints), especially in the right hand. (B) Palmar view of the hands and wrists. Key findings include: 1) thenar muscle atrophy, more pronounced in the left hand, indicative of chronic median nerve compression from carpal tunnel syndrome; 2) acrocyanosis (purplish discoloration) of multiple fingertips (both hands), suggesting compromised peripheral perfusion at the capillary sampling sites.

While distal pulses were present, the fingertips were cool to the touch compared to the forearms or palms and exhibited a slight purplish discoloration (acrocyanosis) (Figure 1). Capillary refill time in the fingertips was assessed and found to be prolonged at >4 seconds bilaterally (normal <2 seconds). Radial and ulnar pulses were palpable bilaterally with normal amplitude. No Allen test was performed. The patient had no history or clinical signs of Raynaud's phenomenon.

The nearly identical bilateral glucose readings (right hand 23 mmol/L, left hand 24 mmol/L) occurred despite asymmetric physical findings: thenar atrophy was more pronounced on the left (correlating with the larger median nerve cross-sectional area of 28 mm²), while osteoarthritic changes were more prominent on the right (KL grade 4 vs. grade 3). This dissociation between the severity of hand pathology and the nearly uniform glucose artifact suggests that the phenomenon, if related to hand pathology, may reflect a systemic predisposition, such as diabetic microvascular disease, rather than strictly local factors, or that a threshold effect exists beyond which further pathology does not increase the artifact (Table 2).

**Table 2:** Relevant Laboratory and Clinical Measurements This table summarizes key laboratory values and objective clinical measurements that contextualize the patient's glycemic control, metabolic status, and severity of hand pathology. The HbA1c of 6.7% one month pre-admission indicates well-controlled diabetes. Hematocrit of 38% is within normal range, excluding low hematocrit as a cause of falsely elevated glucometer readings. Normal renal function (creatinine 0.8 mg/dL, eGFR >60) rules out uremic interference with glucose monitoring. Prolonged capillary refill time (>4 seconds bilaterally) provides objective evidence of compromised peripheral perfusion at the fingertip sampling sites. Median nerve cross-sectional area measurements (right 22 mm², left 28 mm²) confirm severe bilateral carpal tunnel syndrome (normal <10 mm²). Kellgren-Lawrence grading (right grade 4, left grade 3) quantifies the severity of hand osteoarthritis based on radiographic reports. eGFR, estimated glomerular filtration rate; HbA1c, glycated hemoglobin; OA, osteoarthritis.

Parameter	Value	Reference Range
HbA1c (1 month pre-admission)	6.7% (49 mmol/mol)	<6.5%
Hematocrit (admission)	38%	36-46%
Creatinine (admission)	0.8 mg/dL	0.6-1.1 mg/dL
eGFR (admission)	>60 mL/min/1.73m²	>60
Capillary refill time (fingertips)	>4 seconds bilaterally	<2 seconds
Median nerve cross-sectional area (post-discharge)	Right: 22 mm², Left: 28 mm²	<10 mm²
Hand OA Kellgren-Lawrence grade (radiograph reports)	Right: Grade 4, Left: Grade 3	N/A

Follow-up and disposition

The patient's hypoglycemia was promptly treated with oral glucose replenishment, as she was asymptomatic, and she was subsequently stabilized over the next 48 hours with close glucose monitoring using venous samples, along with diet and medication adjustment. She was discharged home on day five with instructions for follow-up with her primary care physician for ongoing diabetes management. The patient was continuously managed by the musculoskeletal physician in the outpatient setting, and she received ultrasound-guided hydrodissection of the median nerve for her symptomatic CTS. Her hand OA, while demonstrating significant deformity, showed improved dexterity following median nerve hydrodissection and was not causing significant pain, requiring no further active intervention. Her initial presenting symptom of knee pain was successfully managed with genicular nerve hydrodissection, which provided effective analgesia without the need for further surgical intervention.

## Discussion

This case report presents a critical, multifactorial failure in diabetic care that originated in an underappreciated anatomical site: the painful, compromised diabetic hand. The near-fatal administration of insulin for undetected hypoglycemia was not due to device malfunction but to a sequential breakdown involving a documented yet paradoxical physiological anomaly, cognitive bias, and protocol-driven inertia. This discussion will dissect these elements to argue that painful hand syndromes may represent an underrecognized threat to the safety of routine point-of-care diagnostics in diabetes, while emphasizing that the observations presented are hypothesis-generating rather than confirmatory.

Systematic exclusion of common error sources

The investigation of this gross discrepancy began with the systematic exclusion of common error sources. Point-of-care glucometer accuracy is vulnerable to a well-documented array of pre-analytical and analytical interferences, including improper calibration, low hematocrit, hypoperfusion, and substance interference [10]. In this case, immediate device recalibration was performed. Furthermore, the error was reproduced using a second glucometer of a different brand, effectively ruling out device-specific malfunction or calibration drift as sole causes. The persistent, bilateral nature of the error across two independent devices confirms a physiological origin specific to the patient.

Systematic exclusion of established causes of pseudohyperglycemia

Before attributing the observed discrepancy to a novel physiological mechanism, we systematically considered and excluded established causes of pseudohyperglycemia.

Fingertip Contamination

The controlled inpatient setting made contamination with glucose-containing substances (e.g., intravenous fluids, food residue) unlikely. The patient had no known exposure to such substances prior to testing, and the reproducibility across multiple sites and times further argues against sporadic contamination.

Hematocrit Effects

Low hematocrit is a well-recognized cause of falsely elevated glucometer readings, as most devices are calibrated for normal hematocrit ranges [10]. The patient's hematocrit was 38%, within the normal range (36%-46%) and within the compensation range for modern glucometers (Table 2).

Interfering Substances

The patient's medication regimen (sitagliptin and metformin) is not known to cause falsely elevated glucometer readings. No other common interferents (e.g., high-dose ascorbic acid, acetaminophen, maltose-containing products, or certain vitamins) were identified in the medication record or by patient history.

Sampling-Related Artifacts

Excessive squeezing of the fingertip during sampling can introduce interstitial fluid and alter readings. However, the artifact was reproduced by different clinicians using a standardized technique, persisted across multiple sites, and was confirmed using a second device of a different brand, findings that make technique-dependent error unlikely.

Device-Specific Malfunction

The use of two independently calibrated glucometers from different manufacturers, both yielding nearly identical readings (Table 1), effectively excludes device-specific malfunction or calibration drift as the primary cause [10].

Site-Specific Factors

The demonstration that arm sampling using the same glucometers produced accurate readings, closely approximating venous glucose, while fingerstick readings remained elevated localizes the artifact to the fingertip itself (Table 1), effectively ruling out systemic metabolic disturbance or generalized assay interference.

Having systematically excluded these common confounders, the error appears to be of physiological origin and specific to the fingertip in the context of this patient's advanced diabetic hand pathology. However, we emphasize that this remains an association, not proven causation, and requires prospective validation.

A profound physiological paradox

This finding presents a profound physiological paradox. The established clinical expectation is that compromised peripheral perfusion, a hallmark of the advanced diabetic hand, leads to pseudohypoglycemia due to increased tissue glucose extraction during slowed capillary transit [11, 12]. Our observation of sustained, bilateral pseudohyperglycemia directly contradicts this canonical model. As highlighted by the patient's physical examination and quantified in Table 2, the coexistence of bilateral CTS with median nerve enlargement (22 mm² right, 28 mm² left), severe hand OA (KL grade 4 right, grade 3 left), palpable subcutaneous fibrosis, and prolonged capillary refill time (>4 seconds) creates a uniquely deranged local milieu [5, 7]. While this complex environment of microvascular dysfunction, chronic inflammation, and interstitial fibrosis is a plausible site for a novel sampling artifact, the specific mechanism driving glucose concentration upward remains unexplained by the current literature [7, 8, 16].

Hypothesis-generating observations

While the precise mechanism remains unexplained, several observations from this case generate testable hypotheses. The site-specific nature of the artifact, normal readings from arm sampling despite persistent fingerstick elevation (Table 1), localizes the phenomenon to the fingertip itself. The nearly identical bilateral glucose readings (right hand 23 mmol/L, left hand 24 mmol/L) occurred despite asymmetric physical findings: thenar atrophy was more pronounced on the left (correlating with the larger median nerve cross-sectional area of 28 mm²), while osteoarthritic changes were more prominent on the right (KL grade 4 vs. grade 3). This dissociation between the severity of hand pathology and the nearly uniform glucose artifact suggests that the phenomenon, if related to hand pathology, may reflect a systemic predisposition, such as diabetic microvascular disease, rather than strictly local factors, or that a threshold effect exists beyond which further pathology does not increase the artifact.

We offer the following as speculative hypotheses requiring direct investigation, not as conclusions. It is possible that chronic inflammation, microvascular dysfunction, and interstitial fibrosis may disrupt normal capillary-interstitial fluid equilibrium, potentially leading to an unrepresentative mixture of capillary blood and sequestered interstitial fluid at the sampling site. The partial reversibility of the artifact following median nerve hydrodissection, with the fingerstick-venous discrepancy improving from +20.5 mmol/L pre-procedure to +1.5 mmol/L at one-month follow-up (Table 1), provides preliminary, though uncontrolled, support for a contributing role of nerve compression and its associated microvascular effects. However, these interpretations remain speculative and require confirmation through prospective studies with objective quantification of tissue pathology and controlled glucose monitoring before and after targeted interventions.

Cognitive and systems factors

However, the physiological anomaly alone did not cause the error; it required uncritical clinical acceptance. This case exemplifies two well-documented cognitive pitfalls in diagnostic safety that can affect patient outcomes. First, automation bias led to the initial trust in the numerically "objective" device reading over the contradictory clinical picture of an asymptomatic patient [13, 17]. Second, protocol-driven inertia created a pathway in which the sliding-scale insulin order became the default next step, requiring deliberate cognitive effort to override [17, 18]. These cognitive biases, including anchoring and confirmation bias, are well-recognized contributors to diagnostic error in clinical medicine, particularly in complex patients with multiple comorbidities [18, 19]. The physician's intervention to order a venous glucose test represents a successful application of "intelligent trust" and a crucial diagnostic pause, the mandatory integration of clinical context with technological data [17, 18]. This step, however, should not rely solely on individual vigilance but must be systematized into safer workflows [16, 17].

Clinical considerations derived from this observation

**Figure 2:**
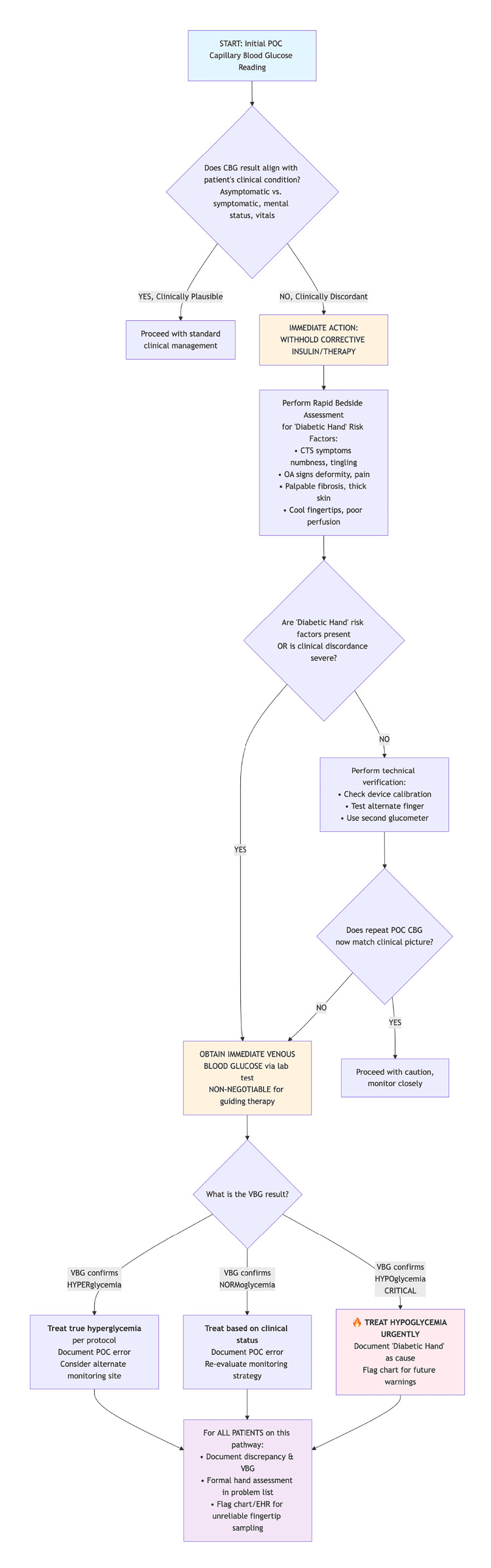
Proposed Clinical Pathway for Managing Discordant Point-of-Care Glucose Readings: A Hypothesis-Generating Framework This algorithm presents a conceptual pathway, derived from a single case observation, for evaluating clinically unexplained point-of-care (POC) capillary blood glucose (CBG) results, particularly in patients with findings suggestive of "diabetic hand" syndrome. This pathway is proposed as a framework for clinical discussion and future research; it has not been validated in prospective studies and should not be interpreted as an evidence-based standard of care. The pathway begins when a POC CBG result appears discordant with the patient's clinical condition. Critical actions include: (1) immediate withholding of corrective insulin therapy; (2) rapid bedside assessment for hand pathology; and (3) consideration of venous blood glucose (VBG) verification when risk factors are present or clinical suspicion remains. The gold-standard VBG result then guides safe management. This conceptual model illustrates how automation bias may be interrupted by embedding a "cognitive pause" into clinical workflow. CBG, capillary blood glucose; CTS, carpal tunnel syndrome; EHR, electronic health record; OA, osteoarthritis; POC, point-of-care; VBG, venous blood glucose. Image credits to Dr. King Hei Stanley Lam.

While this single case cannot establish diabetic hand pathology as a proven risk factor for pseudohyperglycemia, it generates several hypotheses and raises clinical considerations that may warrant attention in appropriate contexts. These are offered not as mandates but as observations for clinicians to consider in light of their own clinical judgment and the limited evidence base. Figure 2 presents a conceptual pathway summarizing these considerations in a structured algorithm.

Consideration of Hand Status in Selected Patients

For clinicians managing diabetes who encounter unexplained discordance between point-of-care glucose readings and clinical status, a brief hand assessment may be considered. Simple screening for symptoms of CTS (nocturnal paresthesia, hand weakness) and signs of OA (joint deformity, pain on motion) can be performed rapidly at the bedside [5, 7]. In this case, such findings were present and associated with a profound monitoring artifact. Whether hand pathology is causally linked to such artifacts, and how commonly this occurs, requires prospective investigation.

Reinforcement of a Fundamental Safety Principle

This case reinforces a well-established safety principle that applies to all point-of-care testing, independent of any specific risk factor: any point-of-care glucose reading that is unexpected or contradicts the patient's clinical state should prompt consideration of verification via venous laboratory glucose before therapeutic action [16]. This principle is not new, nor is it derived from this case alone. It is a cornerstone of safe clinical practice that has prevented iatrogenic errors. The case provides a clear illustration of why this principle matters.

Cognitive Pauses in Clinical Workflow

At an institutional level, electronic health record (EHR) alerts for critical point-of-care values could be designed to include a mandatory "cognitive pause" field [17]. A prompt such as "Does this result match the patient's clinical condition?" can disrupt automated thinking and prompt re-evaluation. This intervention does not depend on establishing diabetic hand as a risk factor; it is a general safety measure applicable to all critical results. This approach does not require rare-event training data and leverages existing EHR infrastructure. It embeds into clinical workflow the same "intelligent trust" principle that averted harm in this case: when technology produces a result that does not align with clinical judgment, the result should be questioned rather than followed without verification.

Future Research Directions

The hypothesis generated by this case, that advanced diabetic hand pathology may, in some patients, produce clinically significant pseudohyperglycemia, requires prospective investigation. Future studies should include (a) systematic assessment of glucose monitoring accuracy in patients with documented CTS and hand OA, (b) objective quantification of hand pathology (e.g., ultrasound, nerve conduction studies, radiographic grading), (c) comparison of fingerstick versus alternative site (e.g., forearm) measurements, and (d) where feasible, evaluation of changes in monitoring accuracy following treatment of hand pathology.

Emerging research on artificial intelligence-assisted clinical decision-making suggests potential applications for integrating comorbidity data with real-time glucose trends to flag patients at risk for monitoring errors [20]. Recent clinical trials have demonstrated the feasibility of AI-assisted insulin titration systems for improving glucose control in patients with type 2 diabetes [21]. However, applying such approaches to rare phenomena like the one described here would require large-scale validation datasets that are not currently available. At present, the most practical and evidence-supported safety measure remains the universal principle of venous verification for clinically discordant results.

Limitations

This report has several important limitations. First, as a single case observation, it cannot establish causality or generalizability. The proposed mechanistic link between diabetic hand pathology and pseudohyperglycemia is hypothesis-generating only and lacks direct physiological or biochemical validation. Alternative explanations, although systematically considered and provisionally excluded, cannot be entirely ruled out in the absence of controlled experimental conditions.

Second, objective quantification of hand pathology was incomplete during the acute admission. While ultrasound measurements of median nerve cross-sectional area and radiographic Kellgren-Lawrence grading were obtained from post-discharge records, nerve conduction studies, the gold standard for CTS diagnosis, were not performed. This limits the precision with which we can correlate disease severity with the observed artifact.

Third, while the temporal association between hydrodissection and improved glucose monitoring accuracy is suggestive (Table 1), the observation is uncontrolled and subject to potential confounding, such as spontaneous resolution, regression to the mean, or unmeasured variables. We cannot definitively attribute the improvement to the intervention.

Fourth, vascular assessment was largely qualitative. Although capillary refill time was measured (>4 seconds), more objective measures such as skin temperature differentials, laser Doppler flowmetry, or formal peripheral vascular studies were not obtained.

Fifth, we did not assess whether the artifact resolved following treatment in a controlled manner with repeated fingerstick-venous comparisons at standardized time points post-intervention, which would have provided a stronger natural experiment supporting a causal link. These gaps should inform the design of future prospective studies investigating this phenomenon.

## Conclusions

In conclusion, this case documents an unexpected and clinically dangerous observation: sustained, bilateral pseudohyperglycemia arising from a limb with clinical signs of hypoperfusion in a patient with advanced diabetic hand syndrome (bilateral CTS with median nerve enlargement of 22-28 mm², Kellgren-Lawrence grade 3-4 hand OA, and palpable fibrosis). Systematic exclusion of common technical errors, including device recalibration, testing with two different glucometers, and bilateral verification, confirmed a reproducible physiological artifact localized to the fingertips, with normal readings obtained from arm sampling using the same devices. This report does not establish diabetic hand pathology as a proven cause of pseudohyperglycemia, nor does it provide evidence for the prevalence of this phenomenon. Rather, it generates a testable hypothesis: that severe microvascular dysfunction, chronic inflammation, and interstitial fibrosis, characteristic of the advanced diabetic hand, may, in some patients, fundamentally disrupt the reliability of fingertip capillary glucose sampling. The site-specific nature of the artifact (normal arm sampling despite persistent fingerstick elevation) and its partial reversibility following median nerve hydrodissection provide preliminary, though uncontrolled, observations that warrant prospective investigation.

The case does, however, reinforce a fundamental and well-established safety principle applicable to all point-of-care testing: when a result contradicts the clinical picture, the result should be verified before action is taken. The physician's decision to obtain venous confirmation, despite a protocol-driven impulse to administer insulin, averted potentially fatal iatrogenic harm. This principle, trust the patient, not just the meter, remains the most reliable safeguard and should be emphasized in clinical training and workflow design. While we propose that heightened awareness of hand pathology may be warranted in patients with unexplained glucose discrepancies, we caution against extrapolating this single observation into routine screening recommendations without supporting epidemiological data. Larger prospective studies are needed to determine the prevalence, risk factors, and mechanisms of this phenomenon before any practice-level changes can be recommended.
